# Retrospective case-control study of intraoperative EEG correlates in postoperative delirium

**DOI:** 10.3389/fmed.2025.1635453

**Published:** 2025-08-13

**Authors:** Longbiao Zhao, Dongjie Qiu, Li Jia

**Affiliations:** ^1^Department of Anesthesiology, The Third Hospital of Hebei Medical University, Shijiazhuang, China; ^2^Department of Anesthesiology, The Fourth Hospital of Hebei Medical University, Shijiazhuang, China

**Keywords:** elderly patient, postoperative delirium, electroencephalogram, α/β-band power, MMSE

## Abstract

**Objectives:**

This retrospective study investigated the association between perioperative SedLine EEG parameters and the incidence of postoperative delirium (POD) in elderly patients undergoing breast cancer surgery.

**Methods:**

A total of 80 elderly patients, aged 60 years or older, who underwent breast cancer surgery between January and December 2024, were included in the analysis. Patients were divided into the POD group and the NPOD group according to whether they developed postoperative delirium. Intraoperative EEG parameters captured by the SedLine monitor included the patient state index (PSI), spectral edge frequency (SEF), burst suppression (BS), and burst suppression ratio (BSR). Raw EEG recordings spanning the entire surgical procedure were preprocessed using EEGLAB. For spectral analysis, power spectral density (PSD) within each frequency band was computed by averaging across all frequency points in that band.

**Results:**

Of the 80 patients, 18 (22.5%) developed POD, while 62 (77.5%) did not. Patients in the POD group had significantly lower preoperative MMSE scores and were less likely to have comorbid hypertension. No significant differences were observed in PSI values between the two groups. However, SEF values (both left and right hemispheres) were significantly lower in the POD group, which also showed a higher incidence of BS. Especially in BSR exceeded 10% for over 1 min. Multivariate logistic regression identified low MMSE scores, absence of hypertension, and decreased SEF-L/SEF-R as independent predictors of POD (*P* < 0.05). Further EEG spectral analysis conducted with MATLAB revealed significantly reduced α-band power (8–13 Hz) in the POD group, especially at frontal electrodes Fp1, Fp2, F7, and F8 (*P* < 0.05), along with diminished β-band power in the same regions.

**Conclusion:**

Lower intraoperative SEF values and reduced α- and β-band EEG power were associated with an increased risk of POD. These EEG markers might serve as early predictors for identifying patients at high risk of developing POD.

## Introduction

Postoperative delirium (POD) and postoperative cognitive dysfunction (POCD) are high-risk neuropsychiatric complications that commonly occur following certain surgical procedures. POD, in particular, is associated with serious consequences, including increased mortality and long-term cognitive impairment. Clinically, delirium may manifest as either hypoactive or hyperactive behavioral disturbances (1–3). POD typically emerges within 24–72 h after surgery. Despite its high incidence, POD remains under recognized in clinical settings, and most affected patients receive insufficient attention or treatment. The consequences of POD are far-reaching, including prolonged hospitalization, significantly increased medical expenses, higher rates of perioperative complications, both short- and long-term, such as postoperative cognitive dysfunction, and in severe cases, adverse prognostic outcomes or permanent neurological impairment (4, 5). With the ongoing rise in population aging, the number of elderly patients undergoing breast cancer surgery continues to grow. Older patients often present with multiple comorbidities, altered internal homeostasis, and increased permeability of the blood-cerebrospinal fluid barrier, all of which heighten their vulnerability to perioperative neurological disorders such as POD.

The SedLine brain function monitor (Masimo, United States) (6) employs multichannel electroencephalogram (EEG) acquisition and spectral analysis to provide a comprehensive assessment of cerebral activity. It captures data including raw EEG signals, burst suppression (BS), burst suppression ratio (BSR), spectral edge frequency (SEF), density spectral array (DSA), patient state index (PSI), and electromyography (EMG). This enables anesthesiologists to interpret cortical function in real-time, optimize anesthetic drug titration, and maintain an appropriate depth of anesthesia, ultimately aiming to reduce the risk of postoperative neurological complications. In our institution, intraoperative EEG monitoring using SedLine has become standard practice.

Previous studies have reported that certain intraoperative EEG features, such as reduced SEF, absence of slow-wave activity during induction, and decreased spectral power during anesthesia, may be linked to the development of POD (7). In this study, we retrospectively analyzed original intraoperative EEG data from elderly patients undergoing breast cancer surgery with SedLine monitoring. Our aim was to compare intraoperative EEG characteristics between those who developed POD and those who did not and to explore potential EEG biomarkers predictive of POD. These findings might serve as a valuable reference for future clinical research and intervention strategies targeting the prevention of POD.

## Materials and methods

### Clinical case data

This study was approved by the Medical Ethics Committee of the Fourth Hospital of Hebei Medical University (Approval No. 2025KS009). As a retrospective, observational study, a waiver of informed consent was granted. The analysis encompassed a total of 80 elderly patients, each aged 60 years or older, who underwent breast cancer surgery in the Fourth Hospital of Hebei Medical University from January to December 2024.

Inclusion criteria for this study were as follows: (1) patients aged 60 years or older; (2) continuous SedLine EEG monitoring throughout the surgical procedure with complete EEG data available; and (3) American Society of Anesthesiologists (ASA) physical status classification I to III.

Exclusion criteria included: (1) emergency surgery or preoperative coma; (2) history of psychiatric or neurological disorders, such as schizophrenia, Parkinson’s disease, epilepsy, or dementia; (3) preoperative cognitive impairment; (4) severe cardiac disease, defined by a preoperative left ventricular ejection fraction below 50%, pacemaker implantation, or presence of an automatic implantable cardioverter-defibrillator (AICD); and (5) advanced renal dysfunction requiring preoperative renal replacement therapy.

### Grouping strategy

Patients were categorized into two groups: the POD group and the non-POD (NPOD) group. The presence of POD was assessed using the Nursing Delirium Screening Scale (Nu-DESC) (Supplementary Table 1).

## Methods

### General clinical and EEG monitoring data collection

Detailed baseline information was collected for both the POD and NPOD groups, including age, ASA physical status classification, years of formal education, preoperative Mini-Mental State Examination (MMSE) scores, and the presence of comorbidities such as hypertension, diabetes mellitus, coronary artery disease, and cerebrovascular disease.

Intraoperative EEG parameters were extracted from the SedLine monitor, including SEF, PSI, and BS. Especially in BSR exceeded 10% for over 1 min. BS refers to a pattern characterized by alternating periods of high-voltage activity and suppression, defined as a voltage amplitude below 10 μV. The BSR represents the proportion of time during which the electroencephalogram remains suppressed within a given time interval. These values were continuously computed by the SedLine system throughout the surgical procedure.

### Original EEG signal acquisition and preprocessing

Continuous raw EEG signals were recorded intraoperatively via a direct data cable connection. The total duration of EEG analysis for each subject spanned approximately 1 h, covering the period from anesthesia induction to the completion of surgery. The data were preprocessed using the EEGLAB toolbox in MATLAB. Electrode placement followed the international 10-5 standard system. The EEG signals were filtered using a 0.5 Hz high-pass and 35 Hz low-pass filter, then segmented into 2 s epochs. The denoising procedure involved multiple steps: a high-pass filter was applied to eliminate low-frequency drift, and a low-pass filter was used to reduce high-frequency noise. In addition, manual review was conducted to exclude segments with excessive noise, and a threshold of ±100 μV was set to remove data points falling outside the acceptable range. Frequency-domain analysis was then performed using MATLAB-based scripts. EEG power spectral density (PSD) was calculated for four canonical frequency bands: Delta (δ: 0.5–4 Hz), Theta (θ: 4–8 Hz), Alpha (α: 8–13 Hz), and Beta (β: 13–35 Hz). Each epoch was zero-padded to 256 data points and subjected to a fast Fourier transform (FFT). The resulting power values were log-transformed (log_10_ scale, expressed in dB/Hz), averaged across all epochs, and the mean power within each frequency band was taken as the final PSD value.

### Statistical analysis

Statistical analyses and image processing were conducted using SPSS version 29.0 and MATLAB. Continuous variables were first assessed for normality using the Kruskal-Wallis test. Variables following a normal distribution were reported as mean ± standard deviation and compared using independent samples *t*-tests. To account for multiple comparisons when analyzing differences across the four EEG channels, the false discovery rate (FDR) method was applied to adjust *p*-values, with a significance threshold set at 0.05. Non-normally distributed variables were presented as median with interquartile range (IQR) and analyzed using the Mann-Whitney U test. Categorical variables were expressed as frequencies and percentages, with group differences assessed by the chi-square test.

This study employed univariate and multivariate logistic regression analyses to identify factors influencing the occurrence of POD. POD incidence was treated as a binary dependent variable. In contrast, patients’ general conditions, including age, ASA physical status, years of education, hypertension, coronary heart disease, diabetes, cerebrovascular disease, preoperative MMSE score, intraoperative PSI, intraoperative SEF-L and SEF-R, BS incidence rate (%), and sustained BSR > 10% for more than 1 min, served as independent variables. Logistic regression was then applied to determine the independent predictors of POD.

## Results

### Study population

Among the 80 enrolled patients, 18 (22.5%) developed POD (POD group), while 62 (77.5%) did not (NPOD group). Compared with the NPOD group, patients in the POD group had significantly fewer years of formal education, lower preoperative MMSE scores, and a lower prevalence of hypertension. No statistically significant differences were observed between the two groups in terms of age, ASA physical status classification, coronary artery disease, diabetes mellitus, or cerebrovascular disease (Table 1). Notably, the prevalence of hypertension was markedly lower in the POD group (55.6% vs. 85.5%). Additionally, the mean MMSE scores were significantly reduced in POD patients, suggesting worse baseline cognitive function (Table 1).

**TABLE 1:** Baseline characteristics and intraoperative electroencephalogram (EEG) parameters of patients.

General conditions	POD (*n* = 18, 22.5%)	NPOD (*n* = 62, 77.5%)	*P*-value
Age (years)	72 (9.5)	73 (7)	0.909
ASA physical status (I/II/III)	1/6/11	3/28/31	0.67
Years of education (years)	4 (3)	5 (3)	0.366
Hypertension (%)	10 (55.6%)	51 (85.5%)	0.018
Coronary heart disease (%)	8 (44.4%)	30 (48.4%)	0.768
Diabetes (%)	10 (55.6%)	36 (58.1%)	0.850
Cerebrovascular disease (%)	9 (50%)	25 (40.3%)	0.465
Preoperative MMSE	27 (1)	27 (1.25)	0.002
Number of intraoperative hypotension	2 (1)	2 (1)	0.743
Intraoperative sufentanil dosage (μg)	35 (20)	35 (20)	0.720
Intraoperative remifentanil dosage (μg)	400 (100)	400 (150)	0.448
Sedlind parameter			
Intraoperative PSI	31 (1.25)	30 (4)	0.771
Intraoperative SEF-L	2.4 (0.95)	9.2 (1.43)	< 0.001
Intraoperative SEF-R	2.75 (3.43)	8.75 (5.83)	< 0.001
BS incidence rate (%)	11 (65%)	39 (63%)	< 0.001
BSR > 10% sustained for more than 1 min incidence rate	5 (27.8%)	12 (19.4%)	< 0.001

### EEG spectral parameters and POD

No significant differences were observed in PSI values between the POD and NPOD groups. However, compared with the NPOD group, patients in the POD group exhibited significantly lower intraoperative values: SEF-L [2.4 (0.95) vs. 9.2 (1.43)] and SEF-R [2.75 (3.43) vs. 8.75 (5.83)]. Additionally, the incidence of BS was significantly higher in the POD group, including the incidence rate of BSR > 10% sustained for more than 1 min (Table 1).

### Multivariate logistic regression analysis

A multivariate logistic regression analysis was conducted using POD occurrence as the dependent variable. Covariates included hypertension, preoperative MMSE score, years of education, intraoperative SEF-L and SEF-R, BS incidence rate, and BSR > 10% sustained for more than 1 min incidence rate. The analysis identified hypertension, lower preoperative MMSE scores, and reduced SEF-L/SEF-R values as independent risk factors associated with the development of POD (*P* < 0.05; Table 2).

**TABLE 2:** Multivariate logistic regression analysis.

Independent variables	OR (95% CI)	*P*-value
Hypertension	0.203 (0.095, 0.703)	0.012
Preoperative MMSE	0.354 (0.145, 0.863)	0.022
Years of education	0.855 (0.635, 1.151)	0.301
Intraoperative SEF-L	0.199 (0.056, 0.710)	0.013
Intraoperative SEF-R	0.577 (0.426, 0.781)	< 0.001
BS incidence rate (%)	1.168 (0.302, 4.508)	0.822
BSR greater than 10% and sustained for more than 1 min (%)	1.474 (0.347, 4.250)	0.599

### DSA analysis

**FIGURE 1:**
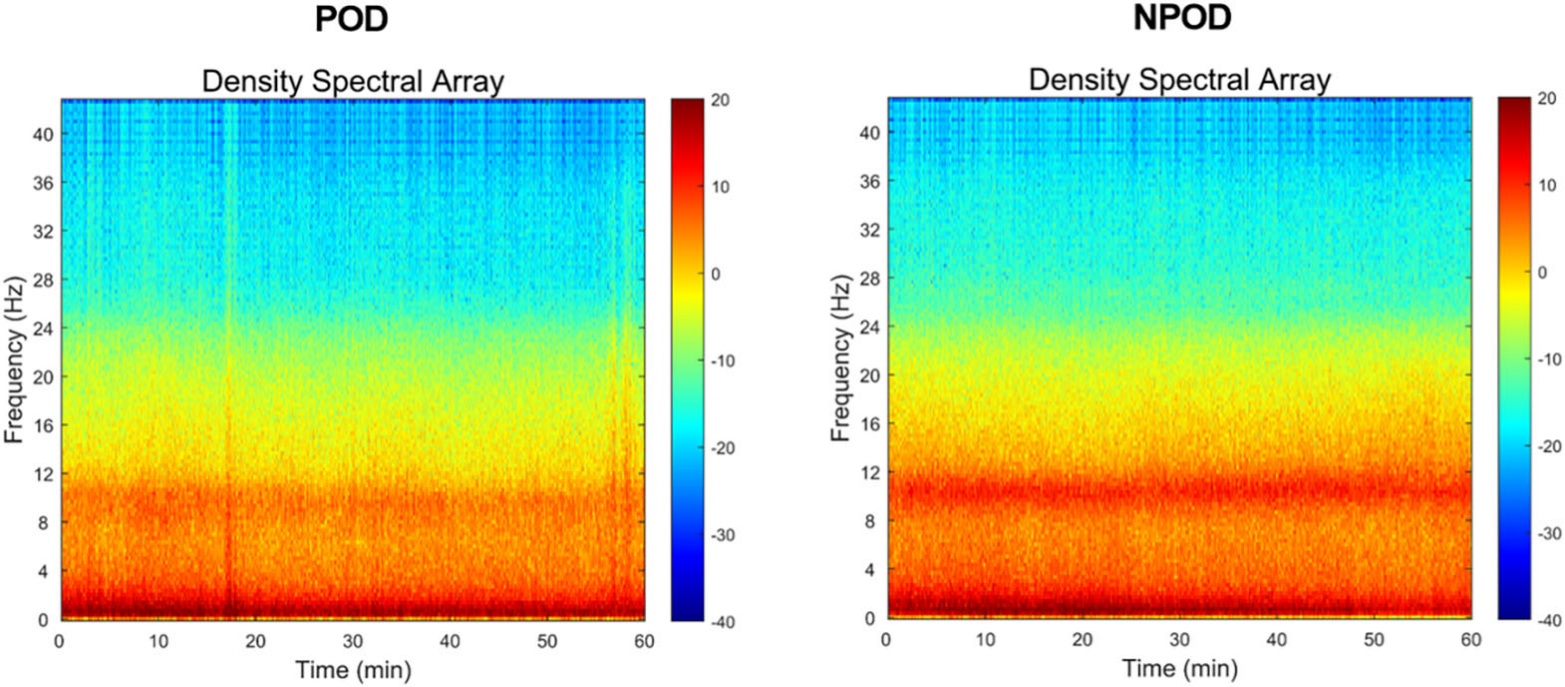
In the density spectral array (DSA), time (min) is arranged along the x-axis, and frequencies (Hz) are arranged along the y-axis. Frequency within the δ-band (0.5∼4 Hz)/θ-band (4∼8 Hz)/α-band (8∼13 Hz) of the postoperative delirium (POD) group was reduced, compared to the non-postoperative delirium (NPOD) group.

Density spectral array results revealed a noticeable reduction in α-band (8–13 Hz) power in patients who developed POD, with significant attenuation observed in multiple cortical regions (Figure 1).

### PSD analysis

**FIGURE 2:**
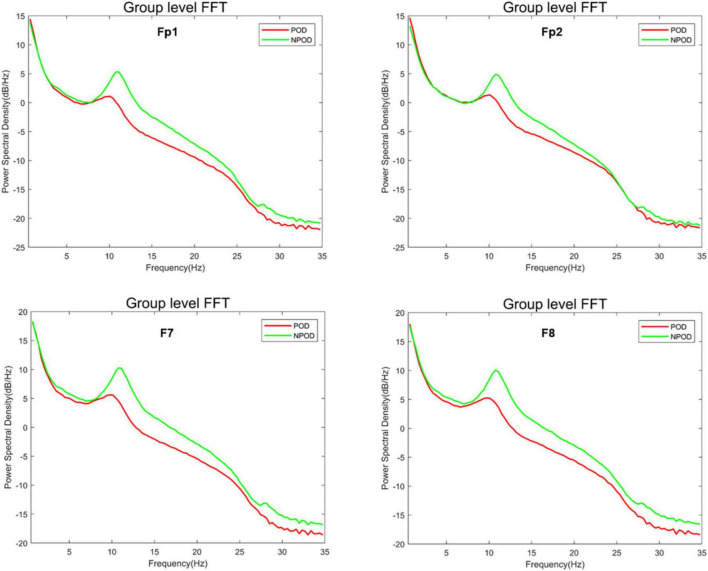
The difference in power spectral density (PSD) between the postoperative delirium (POD) group and the non-postoperative delirium (NPOD) group of the different channels (Fp1/Fp2/F7/F8).

Using a custom MATLAB script (MathWorks Inc.), we computed the median differences in power across frequencies to assess intergroup differences in EEG spectral characteristics. Compared to the NPOD group, patients in the POD group demonstrated overall lower PSD across all channels. Specifically, PSD within the α-band (8–13 Hz) and β-band (> 13 Hz) was significantly reduced in the POD group (Figure 2).

### Differences in EEG power across frequency bands between groups

**FIGURE 3:**
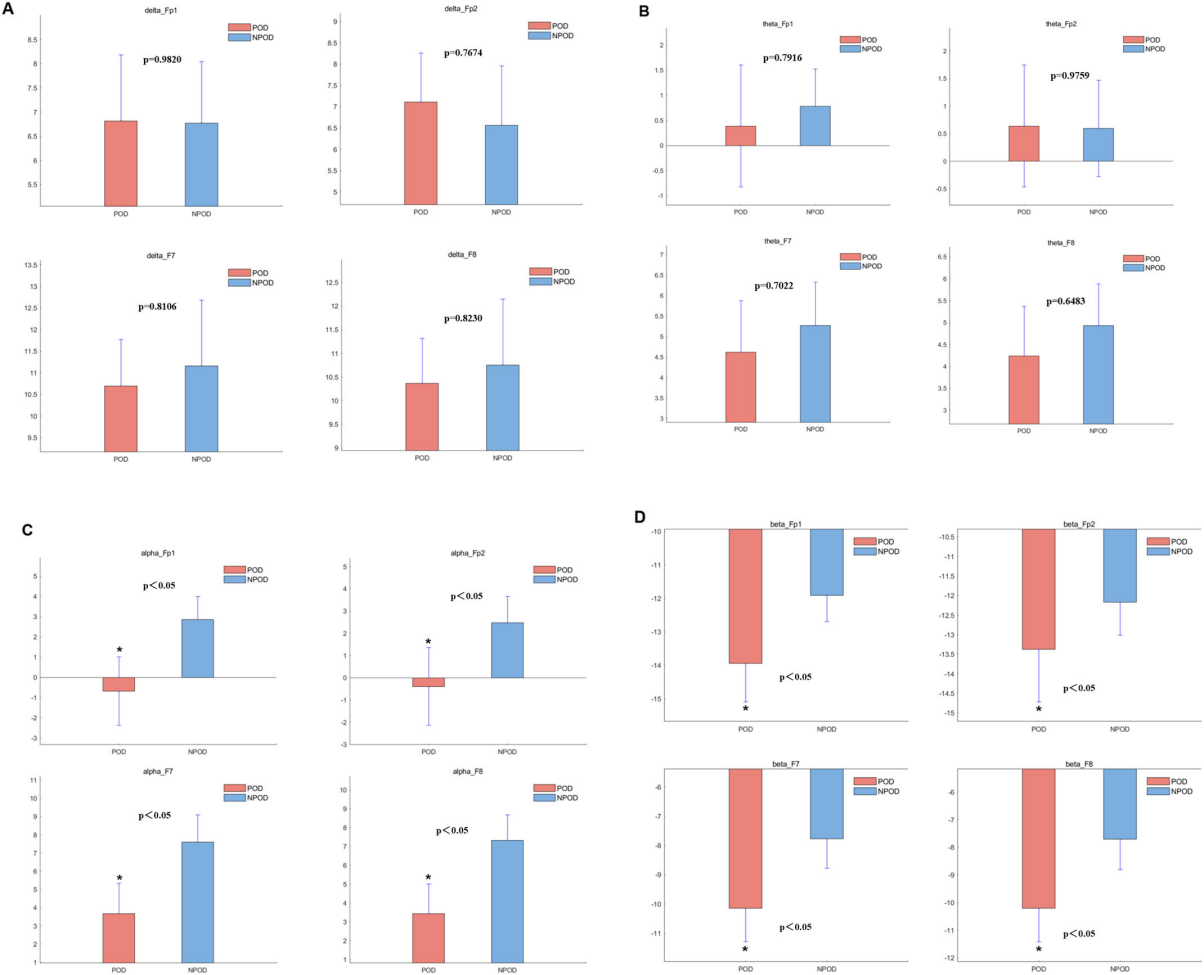
The difference in power within the different channels between the postoperative delirium (POD) group and the non-postoperative delirium (NPOD) group. **(A)** δ-band (0.5∼4 Hz); **(B)** θ-band (4∼8 Hz); **(C)** α-band (8∼13 Hz); **(D)** β-band (> 13 Hz).

The δ-band power in the POD group was lower than in the NPOD group at the F7 and F8 electrode sites; however, this difference did not reach statistical significance (Figure 3A). Similarly, θ-band power was reduced in the POD group compared to NPOD at Fp1, F7, and F8 channels, but these differences were not statistically significant (Figure 3B). In contrast, α-band power was significantly decreased in the POD group at Fp1, Fp2, F7, and F8 channels (*P* < 0.05) (Figure 3C). Likewise, β-band power in the POD group was significantly lower than in the NPOD group at the same electrode sites (Fp1, Fp2, F7, F8) (*P* < 0.05) (Figure 3D).

## Discussion

Although previous univariate analyses have associated POD with advanced age, fewer years of education, and lower MMSE scores (8–11), no such correlations were observed in this retrospective case-control study. Nonetheless, our results reaffirmed the predictive value of preoperative MMSE scores in identifying patients at elevated risk for POD. Notably, the multivariate logistic regression revealed that hypertension and preoperative MMSE were independently associated with POD occurrence.

Contrary to some earlier studies, we did not observe a significant association between years of education and POD in either univariate or multivariate analyses, which might reflect limitations related to sample size and warrants further investigation with larger cohorts. Interestingly, fewer patients in the POD group had hypertension, raising questions about whether this might correlate with less stringent intraoperative blood pressure management, potentially contributing to an increased risk of postoperative cognitive complications. Although some literature suggests that intraoperative hypotension is a risk factor for POD, for instance, elderly males undergoing laryngectomy with low education levels and prolonged surgery duration (12, 13), other studies, including a retrospective cohort analysis, have found no such association in elderly patients undergoing elective non-cardiac surgery (14). We found no evidence to support this conclusion, as this study did not include a statistical analysis of blood pressure control levels between the two groups. Therefore, confirmation through a well-designed prospective study with a larger sample size is warranted in the future.

Patient status index parameters did not differ significantly between the POD and NPOD groups. However, intraoperative SEFs (SEF-L and SEF-R) were markedly lower in POD patients compared to those without POD. Additionally, the incidence of BS was significantly higher in the POD group, with an increased occurrence of BSR exceeding 10% and persisting for more than 1 min. These findings aligned with the study by Soehle et al. (15), which demonstrates that intraoperative BS assessment can help identify patients at risk for POD. Furthermore, the duration of BSR, as determined by visual EEG analysis, has been significantly correlated with POD incidence (16), corroborating our results. Our multivariate logistic regression analysis also confirmed that reduced intraoperative SEF-L and SEF-R values were independently associated with an increased risk of POD.

Density spectral array analysis revealed a reduction in α-band (8–13 Hz) power in the POD group. Similarly, PSD analyses showed that α-band and β-band (> 13 Hz) power were decreased in POD patients compared to NPOD patients, particularly in the Fp1, Fp2, F7, and F8 channels. These findings were consistent with those from a prospective, single-center observational study reporting decreased α- and β-band power and lower SEF values in patients with POD (17). Although δ-band power at F7 and F8 and θ-band power at F8 were lower in the POD group, these differences did not reach statistical significance, which might be attributed to the relatively small sample size. Purdon et al. have demonstrated that general anesthetics increase alpha power in the frontal region (18). Koponen et al. (19) have analyzed the EEG spectrum during delirium episodes, revealing elevated delta/theta power alongside reduced alpha power compared to cognitively normal subjects. Ching et al. (20) have proposed that anesthesia-induced alpha oscillations arise from reverberations within a thalamo-cortical loop, likely driven by thalamic hyperpolarization. Gutierrez’s findings suggest that the absence of increased alpha power during anesthesia may result from thalamo-cortical circuitry dysfunction, potentially underlying the low alpha power associated with POD (21). Our findings were consistent with these studies; however, confirmation through future research with adequate sample sizes is necessary. Overall, reduced SEF values and diminished α- and β-band power appeared to be promising EEG biomarkers for early identification of patients at elevated risk of developing POD during anesthesia.

Baseline EEG frequencies naturally decline with aging, as elderly individuals exhibit reduced spectral EEG activity during wakefulness compared to younger populations (22). In our study, although there was no significant age difference between the POD and NPOD groups, overall EEG frequencies were diminished and preoperative MMSE scores were lower in the POD group. This suggested that the observed EEG frequency differences were attributable not to chronological age but rather to the patient’s underlying functional brain status. Supporting this, Musaeus et al. (23) have also reported that mild cognitive decline in older adults is associated with slower EEG frequencies. Therefore, it was reasonable to infer that the preexisting cognitive impairment in our POD patients might underlie the reduced intraoperative SEF values and decreased α/β-band power.

We concluded that lower preoperative MMSE scores, reduced intraoperative SEF values, and diminished α/β-band power constituted significant risk factors for POD.

## Limitations

Our study was limited by a relatively small sample size. Additionally, frequent EEG artifacts led to heterogeneity in the analyzed EEG time points. The EEG data analyzed were restricted to a 1 h intraoperative window, without assessment of preoperative or postoperative EEG changes. Moreover, POD identification relied solely on the Nu-DESC. The Nu-DESC is simple and easy to administer, but cannot definitively diagnose POD. The Confusion Assessment Method (CAM) and its ICU-adapted version (CAM-ICU) offer high sensitivity (94%–100%) and specificity (90%–95%), with rapid and straightforward evaluation; however, these tools are limited to diagnosing POD and do not effectively assess its severity. To grade POD severity, instruments such as the Delirium Rating Scale–Revised-98 or the Memorial Delirium Assessment Scale can be employed. Since this is a retrospective study, prior patient data included only Nu-DESC scores, without CAM or CAM-ICU assessments.

Given the variability in sensitivity and specificity across delirium assessment tools, future studies should consider combining Nu-DESC with the CAM or Delirium Rating Scale-Revised-98 to enhance diagnostic accuracy.

## Conclusion

Reduced intraoperative SEF values and decreased α/β-band power were associated with an increased risk of POD. These EEG parameters held promise as early, objective predictors to identify patients at heightened risk for developing POD.

## Data Availability

The original contributions presented in this study are included in this article/Supplementary material, further inquiries can be directed to the corresponding authors.
