# Supplementary material for: Retrospective case-control study of intraoperative EEG correlates in postoperative delirium
Source: Front Med (Lausanne). 2025 Aug 13;12:1635453. doi: 10.3389/fmed.2025.1635453 (PMC12352117; doi:10.3389/fmed.2025.1635453)
Supplement: Supplementary file 1 [file Table_1.pdf]

Supplement 1 Nursing delirium screening score (Nu-DESC).

Symptom	Symptoms Rating (0-2)	Score
I. Disorientation	Verbal or behavioural manifestation of not being oriented to time or place or misperceiving persons in the environmen	□ 0<br>□ 1<br>□ 2
II. Inappropriate behaviour	Behaviour inappropriate to place and/or for the person; e.g., pulling at tubes or dressings, attempting to get out of bed when that is contraindicated, and the like.	□ 0<br>□ 1<br>□ 2
III.Inappropriate communication	Communication inappropriate to place and/or for the person; e.g., incoherence, noncommunicativeness, nonsensical or unintelligible speech.	□ 0<br>□ 1<br>□ 2
IV.Illusions/Hallucinations	Seeing or hearing things that are not there; distortions of visual objects.	□ 0<br>□ 1<br>□ 2
V.Psychomotor retardation	Delayed responsiveness, few or no spontaneous actions/words; e.g., when the patient is prodded, reaction is deferred and/or the patient is unarousable.	□ 0<br>□ 1<br>□ 2
Total score		

Each symptom is recorded on a scale of 0 to 2 points based on its severity: 0 indicates no symptoms, 1 indicates mild symptoms, and 2 indicates severe symptoms. The highest possible score is 10 points, and a total score of 2 points or higher would result in a diagnosis of postoperative delirium.
